# In-Person Versus Telehealth Setting for the Delivery of Substance Use Disorder Treatment: Ecologically Valid Comparison Study

**DOI:** 10.2196/34408

**Published:** 2022-04-04

**Authors:** Quyen M Ngo, Jacqueline E Braughton, Kate Gliske, Lance A Waller, Siara Sitar, Danielle N Kretman, Hannah L F Cooper, Justine W Welsh

**Affiliations:** 1 Butler Center for Research Hazelden Betty Ford Foundation Center City, MN United States; 2 Department of Biostatistics and Bioinformatics Emory University Rollins School of Public Health Emory University Atlanta, GA United States; 3 Department of Psychiatry and Behavioral Services Emory University School of Medicine Emory University Atlanta, GA United States; 4 Department of Behavioral, Social, and Health Education Sciences Emory University Rollins School of Public Health Emory University Atlanta, GA United States

**Keywords:** telehealth, substance use treatment, patient outcomes

## Abstract

**Background:**

The COVID-19 pandemic has profoundly transformed substance use disorder (SUD) treatment in the United States, with many web-based treatment services being used for this purpose. However, little is known about the long-term treatment effectiveness of SUD interventions delivered through digital technologies compared with in-person treatment, and even less is known about how patients, clinicians, and clinical characteristics may predict treatment outcomes.

**Objective:**

This study aims to analyze baseline differences in patient demographics and clinical characteristics across traditional and telehealth settings in a sample of participants (N=3642) who received intensive outpatient program (IOP) substance use treatment from January 2020 to March 2021.

**Methods:**

The *virtual IOP (VIOP) study* is a prospective longitudinal cohort design that follows adult (aged ≥18 years) patients who were discharged from IOP care for alcohol and substance use–related treatment at a large national SUD treatment provider between January 2020 and March 2021. Data were collected at baseline and up to 1 year after discharge from both in-person and VIOP services through phone- and web-based surveys to assess recent substance use and general functioning across several domains.

**Results:**

Initial baseline descriptive data were collected on patient demographics and clinical inventories. No differences in IOP setting were detected by race (*χ^2^_2_*=0.1; *P*=.96), ethnicity (*χ^2^_2_*=0.8; *P*=.66), employment status (*χ^2^_2_*=2.5; *P*=.29), education level (*χ^2^_4_*=7.9; *P*=.10), or whether participants presented with multiple SUDs (*χ^2^_8_*=11.4; *P*=.18). Significant differences emerged for biological sex (*χ^2^_2_*=8.5; *P*=.05), age (*χ*^2^_6_=26.8; *P*<.001), marital status (*χ*^2^_4_=20.5; *P*<.001), length of stay (*F*_2,3639_=148.67; *P*<.001), and discharge against staff advice (*χ*^2^_2_=10.6; *P*<.01). More differences emerged by developmental stage, with emerging adults more likely to be women (*χ*^2^_3_=40.5; *P*<.001), non-White (*χ*^2^_3_=15.8; *P*<.001), have multiple SUDs (*χ*^2^_3_=453.6; *P*<.001), have longer lengths of stay (*F*_3,3638_=13.51; *P*<.001), and more likely to be discharged against staff advice (*χ*^2^_3_=13.3; *P*<.01).

**Conclusions:**

The findings aim to deepen our understanding of SUD treatment efficacy across traditional and telehealth settings and its associated correlates and predictors of patient-centered outcomes. The results of this study will inform the effective development of data-driven benchmarks and protocols for routine outcome data practices in treatment settings.

## Introduction

### Background

In 2019, an estimated 20.4 million individuals aged ≥12 years met the criteria for a substance use disorder (SUD) in the United States. Substance misuse and use remain the leading causes of disability, years of life lost, and death [1,2]. Drug-related overdoses in the United States were responsible for >100,000 deaths in a 12-month period from April 2020 to April 2021 [3-5]. Research supports the idea that the COVID-19 pandemic has further exacerbated the substance use and drug overdose crisis in the United States. Provisional public health data illustrate that drug-related overdose deaths increased by 28.3% from 2019 to 2020 and subsequently by 28.5% from 2020 to 2021 [5]. A study found that 13.3% of US adults reported starting or increasing substance use to cope with pandemic-related stressors and emotions [6]. However, in any given year, <15% of individuals who need specialized substance use–related treatment receive it, illuminating the significant unmet need for substance use–related treatment services in the United States [3].

Novel applications of treatment are necessary to enhance access to care and reduce health care disparities. Before the pandemic, telehealth platforms were already growing in popularity among mental health providers and demonstrated similar treatment outcomes as in-person care [7]. The use of telehealth for the treatment of SUD has historically been lower than its use for general mental health conditions, often focused on individual digital recovery tools and applications [8-10]. A number of barriers exist that largely prevent widespread use including regulations, reimbursement issues, and usability of platforms [11]. The COVID-19 pandemic has created a catalyst for the rapid expansion of SUD services through telehealth platforms. Emergency federal and state policies removed geographic and site-of-service restrictions while increasing the number of telehealth services covered by insurers. Many states also expanded take-home services for methadone, allowed buprenorphine prescriptions without face-to-face requirements, and dropped prior authorization requirements for opioid use disorder medications.

Despite a nationwide increase in telehealth services within licensed substance use treatment facilities, little is known about the long-term effectiveness of substance use interventions delivered through digital technologies [12,13]. Preliminary evidence supports high user engagement across a variety of digital platforms but does not provide a strong evidence base for recovery-related outcomes [10]. Since the onset of the COVID-19 pandemic, the literature has highlighted the need for specific research considerations related to the delivery of telehealth for SUDs, including whether treatment outcomes are comparable between in-person and telehealth delivery methods [14]. Although scholars have contributed to this gap in knowledge, these studies of SUD telehealth have been primarily limited to samples and settings, including a large reliance on single clinics or populations with limited geographic scope that precludes comparisons across national variations. Furthermore, much has been focused on individual treatment formats, whereas even less data exist for telehealth group-based intensive SUD treatment services [15].

### Objectives and Hypotheses

The primary aim of this study is to examine treatment-related outcomes and patient predictors of treatment effectiveness across traditional in-person and telehealth settings in an outpatient addiction treatment setting. This study also aims to better understand the correlates of treatment efficacy in telehealth group formats as well as how outcomes of data collection practices may differentially impact response rates in web-based programming. Finally, this study aims to identify clinician-level characteristics that contribute to the successful engagement of patients and whether these characteristics are also associated with enhanced patient outcomes. The findings of the study provide actionable evidence to sustain internet-based SUD services and offer data to guide an effective response to the SUD public health crisis. The authors hypothesized that patient outcomes and treatment effectiveness across in-person and web-based settings would be comparable while providing equitable patient access across rural and urban geographic locations.

## Methods

### Ethics Approval

This study was evaluated by Emory University’s Institutional Review Board (Emory IRB ID: STUDY00001822) and was determined to have met human research exemption under 45 Code of Federal Regulations 46.104(d; 4), as all study data were collected in the context of Hazelden Betty Ford Foundation’s (HBFF) standard routine outcome monitoring (ROM) practices. Similar to other health care organizations, HBFF’s putative ROM practices include regular, methodical collection of diagnostics, patient progress, and overall treatment effectiveness data beginning at intake and ending 12 months after treatment discharge. The intent of ROM data is to provide direct care providers with consistent, reliable assessments of individual patient progress and treatment experience to reduce instances of treatment deterioration and failure and thereby bolster patient outcomes [16-18]. Similarly, these data inform patient-centered clinical operations and organizational quality improvement procedures to ensure that ethical quality health care is delivered [19,20].

### Study Design and Procedures

The virtual intensive outpatient program (VIOP) study is a naturalistic, prospective longitudinal cohort design that followed patients discharged from IOP care for alcohol and substance use–related treatment at the largest nonprofit treatment provider for SUDs in the United States between January 2020 and March 2021. The HBFF provides SUD treatment for thousands of patients each year through its 17 locations nationwide. In 2019, HBFF began piloting a single VIOP group to better understand the core functionality and acceptability of using a new web-based platform, with an incremental expansion of VIOP planned to begin in 2020. However, the onset of the COVID-19 pandemic greatly accelerated the internet-based rollout, necessitating immediate changes to in-person programming in March 2020. As a result, the HBFF quickly pivoted most IOP services to a web-based format while continuing to collect routine patient outcome data. Within a 2-week period beginning at the end of March, 74 IOP groups transitioned from in-person to web-based programming, representing 541 unique patients.

VIOP was developed to be as close a corollary to in-person IOP as possible while simultaneously expanding access to care to those who may not otherwise have lived close enough to a physical HBFF location to regularly attend the sessions. This included video-based, real-time group interactions and individual sessions, leveraging the use of technology that could accommodate low-bandwidth internet connections, thus ensuring the quality and stability of video feeds during sessions. In-person systems for patient accountability have also been adapted for internet-based care, including crisis or emergency response protocols, privacy monitoring, and random drug and alcohol testing using in-home testing kits or blood alcohol content devices with video support. Remote testing through laboratories in patient communities was also used when needed through a partnership with a remote testing company.

Individuals participating in VIOP who were identified as potentially benefiting from medication for SUD were either partnered with an HBFF provider for evaluation and follow-up or were recommended to obtain a local community provider educated in addiction treatment. Web-based clinical staff were trained to collaborate on treatment recommendations and to provide monitoring to ensure safe use and medication compliance. This monitoring was multifactorial and included increased toxicology, prescription drug monitoring reviews, and multidisciplinary case reviews.

**Figure 1:**
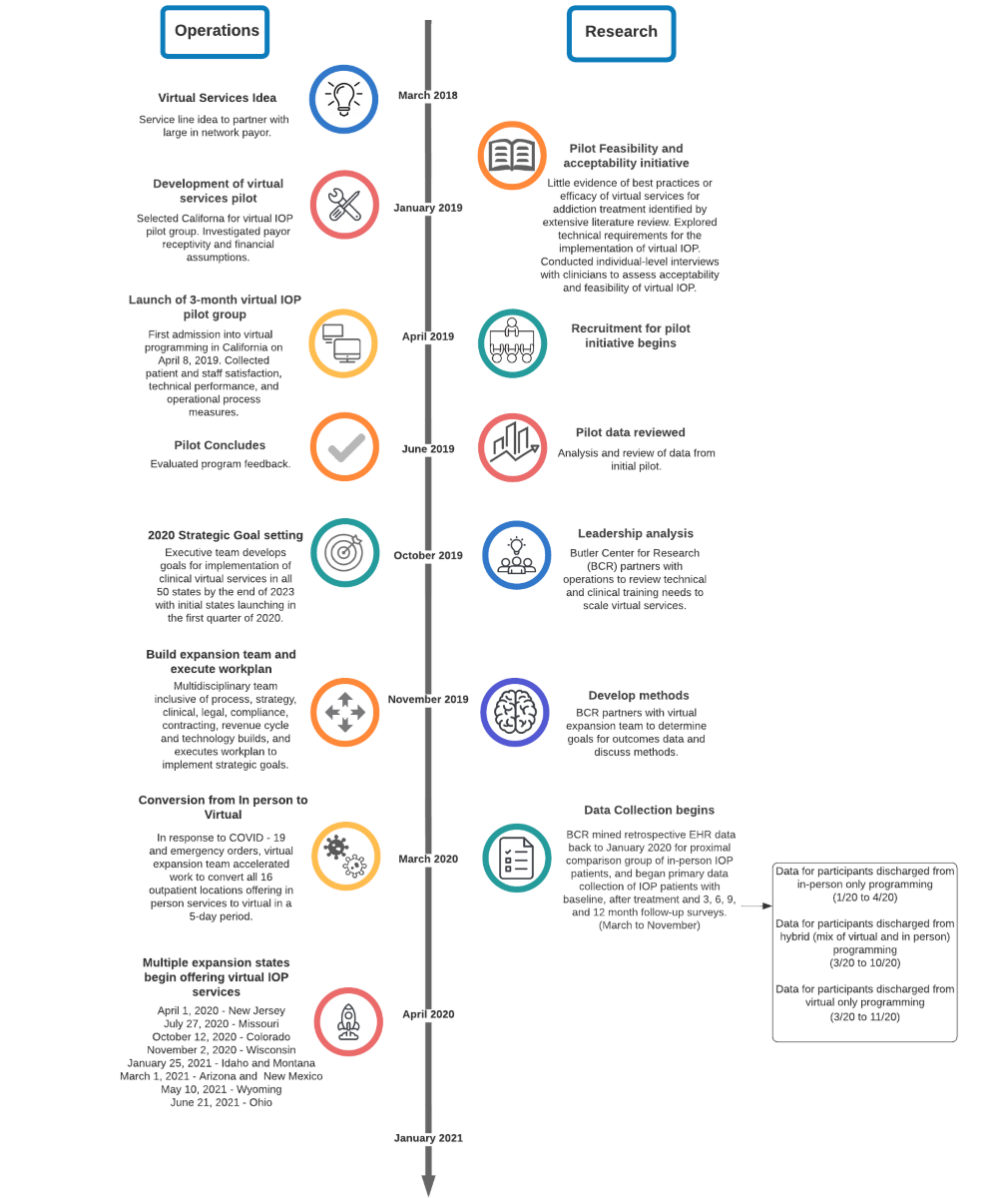
Timeline of development and implementation of web-based services. The left side of the figure depicts the conceptualization and delivery of internet-based services. The right side represents the underlying research and data collection across the protocol timeline. EHR: electronic health record; IOP: intensive outpatient program.

An overview of the operations and study procedures is presented in Figure 1. All patients who were discharged from the IOP between January 1, 2020, and March 17, 2021, were considered eligible and contacted to participate. Although the transition to VIOP occurred at the end of March 2020, study data collection did not begin until May 2020. To capture a comparison group of those who attended IOP in person, all patients who were discharged from any in-person IOP on or after January 1, 2020, were opted to receive the IOP-specific outcome surveys. Owing to the timing of the mass opt-in and the shorter windows of the initial surveys (ie, 30 days from treatment admission for the baseline survey and 30-60 days following discharge for the 1-month survey), most of the in-person and hybrid cohorts were not eligible to complete the baseline or 1-month postdischarge survey.

Data were collected at six periods: baseline (within 30 days of admission) and 1, 3, 6, 9, and 12 months after discharge from IOP, through phone- and web-based surveys. Demographic data and IOP episode-level information, including length of stay, discharge status, and the number of sessions attended, were acquired from *Compass*, HBFF’s electronic health record database management system. The baseline and postdischarge follow-up surveys asked patients to assess their recent substance use and general functioning across several domains, including previous treatment, craving level, peer-support group attendance, use of anticraving medications, substance use, quality of life, economic stressors, exposure to violence, psychological well-being, self-efficacy, gratitude, parental substance use, and parenting stressors.

Baseline and postdischarge follow-up surveys were administered by the HBFF’s team of data collection specialists (DCSs). Each DCS team member was systematically and rigorously trained to follow the same set of procedures to ensure data integrity and security in adherence to patient confidentiality standards as per Health Insurance Portability and Accountability Act and 42 CFR Part 2 governance. As part of the ROM procedures, coordination of survey administration and completion includes a brief check-in between DCS and patients’ primary clinical staff to alert patients of survey availability.

### Study Population

Patients were considered eligible to participate if they were discharged from the IOP at HBFF between January 2020 and March 2021 and were aged ≥18 years at the time of treatment admission. No additional exclusion criteria were applied.

The final sample included 3642 patients who fell into three comparison groups: (1) those who received in-person only programming (957/3642, 26.28%), (2) those who received hybrid in-person and internet-based programming (541/3642, 14.85%), and (3) those who received internet-only programming (2144/3642, 58.87%).

### Measures

#### Overview

Demographics were captured at the time of admission within HBFF’s electronic health records.

The following measures were used to collect data across the 6 periods (Table 1).

**Table 1:** Study measures and time points.

Study measures	Time points					
	Before discharge	After discharge				
	Baseline	1 month	3 months	6 months	9 months	12 months
The System Usability Scale		✓				
Flourishing Scale	✓	✓	✓	✓	✓	✓
Consumer Financial Protection Bureau Financial Well-being Scale—abbreviated	✓	✓	✓	✓	✓	✓
The Gratitude Questionnaire—6-item Form	✓	✓	✓	✓	✓	✓
Patient Health Questionnaire-9	✓					
General Anxiety Disorder-7	✓					
Commitment to Sobriety Scale-5	✓					
Desires for Alcohol Questionnaire-6	✓					
World Health Organization Quality of Life-Brief	✓	✓	✓	✓	✓	✓
Self-efficacy of Sustained Sobriety Scale	✓	✓	✓	✓	✓	✓
12-step peer group engagement	✓	✓	✓	✓	✓	✓
Parenting Daily Hassles Scale	✓	✓	✓	✓	✓	✓
Modified Children of Alcoholics Screening Test-6	✓	✓		✓		✓
Revised Conflict Tactics Scale	✓	✓	✓	✓	✓	✓
Drug and alcohol use	✓	✓	✓	✓	✓	✓

#### Depression Symptoms

Patients were administered the 10-item Patient Health Questionnaire-9 (PHQ-9) to self-report any occurrence of the 9 depression-related symptoms representative of Diagnostic and Statistical Manual of Mental Disorders Volume 5 (DSM-5) Major Depressive Disorder [21]. Each patient was asked to evaluate and rate the frequency of each symptom statement using one of four ordinal categories: 0 (*not at all*) to 3 (*nearly every day*). Possible scores ranged from 0 to 27, with higher scores indicating a greater frequency and severity of the DSM-5 Major Depressive Disorder symptoms. The PHQ-9 had high internal reliability (Cronbach *α*=.89) [21].

#### Anxiety Symptoms

To measure symptoms indicative of co-occurring generalized anxiety disorder (GAD), patients were administered the 8-item General Anxiety Disorder-7 (GAD-7) screener [22]. Patients were asked to reflect on and estimate the occurrence of symptoms indicative of GAD in the past 2 weeks on a 4-point scale, ranging from 1 to 4. Sample questions included, “Over the last two weeks, how often have you been bothered by the following problems? Feeling nervous, anxious, or on the edge.” The response categories included 4 Likert-type ranges of occurrence: 1 (*not at all*), 2 (*several days*), 3 (*more than half the days*), and 4 (*nearly every day).* Patient self-reports were scored and totaled; item scoring replaced the 1-4 scale with designated scores of 0, 1, 2, and 3. Scores range from 0 to 21, with higher scores indicating an increased occurrence and severity of general anxiety symptoms that impede daily functioning. Internal consistency calculations indicated high internal reliability (Cronbach *α*=.82) [22].

#### Confidence and Commitment to Staying Sober

The ratings of patient-perceived level of motivation and dedication to achieving initial and maintaining ongoing sobriety for substance use were measured using the 5-item Commitment to Sobriety Scale (CSS-5) [23]. Each statement was rated on a 6-point Likert scale, ranging from 1 (*strongly disagree*) to 6 (*strongly agree*). Example items included, “Staying sober is the most important thing in my life” and “I will do whatever it takes to recover from my addiction.” The CSS-5 displayed high internal reliability at posttreatment follow-up (Cronbach *α*=.89) [23].

After the completion of the CSS-5, patients were asked to reflect and rate their level of confidence in their commitment to abstinence for the next 30 days using a 10-point scale, ranging from 1 (*not at all confident*) to 10 (*very confident*).

#### Desire and Intent to Use

The 6-item short form Desire for Alcohol Questionnaire was used to assess patient-reported desire (ie, cravings), intent to use, and the role of negative reinforcement on their primary substance of choice [24]. The word *alcohol* in the questionnaire was replaced with *drugs* to expand the measurements’ applicability to a wide range of substances. Patients rated their level of agreement with each statement on a 7-point Likert scale, ranging from 1 (*strongly disagree*) to 7 (*strongly agree*). Statements are comprised two subscales: (1) desire and intention to use (ie, “I want to use drugs so much I can taste it” and “My desire to use drugs now seems overwhelming”) and (2) negative reinforcement (ie, “I would feel as if all the bad things in my life had disappeared if I used drugs now” and “I would feel less worried about my daily problems if I used drugs now”). For each subscale, responses were summed and divided by 3, resulting in a score between 1 and 7, with 7 indicating higher levels of desire and intent to use [24]. Subscale internal reliability calculations illustrated high consistency for both subscales: desire and intention to use (Cronbach *α*=.94) and negative reinforcement (Cronbach *α*=.89) [24].

#### Web-Based Therapy Platform Evaluations

The 10-item System Usability Scale was used to measure patient-perceived evaluations of the usability of the software platform for the VIOP platform [25]. To better fit the applied context, the word *system* was changed to *VIOP platform*, and the word *cumbersome* was replaced with *awkward*. These minor adaptations had no adverse impact on the internal consistency of the scale (Cronbach *α*=.89). Patients were asked to assess and rate their level of agreement with a list of statements describing the usability of the VIOP platform using a 5-point Likert scale, ranging from 1 (*strongly disagree*) to 5 (*strongly agree)*. Example questions included, “I thought the virtual IOP platform was easy to *use*” and “I felt very confident using the virtual IOP platform.”

#### Psychological Well-being

The self-reported Flourishing Scale is a measure of *psychological well-being* comprising 8 statement items indicative of predictors of social–psychological prosperity (ie, flourishing), such as social support, self-acceptance and capability, and leading a purposeful life [26]. As an example, item 5 reads, “I am competent and capable in the activities that are important to me*.”* Patients were instructed to rate their level of concurrence with each item using a 7-point Likert rating scale. Responses were scored from 1 (*strongly disagree*) to 7 (*strongly agree*) and added together to provide a composite score, with higher scores indicating high social–psychological prosperity. Calculations of the scale’s Cronbach *α* showed a high internal consistency (Cronbach *α*=.92).

#### Financial Well-being

Patient-perceived financial well-being, the belief that one is financially secure (ie, can meet current financial commitments) and has financial freedom (ie, the ability to make financial choices that go over and beyond purely basic needs), was assessed using the Consumer Financial Protection Bureau (CFPB) Financial Well-being Scale [27]. Applying a 5-point Likert scale, patients rated their level of agreement with 5 statements describing diverse financial situations. We adapted and recoded the response categories for items 1 through 3 from 5 (*completely)* to 1 (*not at all*) to 4 (*strongly disagree*) to 0 (*strongly agree*) for internal scale validity and reliability. Example item statements included, “Because of my money situation, I feel like I will never have the things I want in life” and “I have money left over at the end of the month.” The alpha calculations indicated high internal consistency (Cronbach *α*=0.88). Higher scores indicated greater perceived financial well-being.

#### Attitudes of Gratitude

Patients’ self-reported propensity toward attitudes of gratitude in day-to-day experiences was assessed using the Gratitude Questionnaire—6-item form [28]. Questionnaire Likert-type responses allowed patients to choose their level of agreement from 1 (*strongly disagree*) to 6 (*strongly agree*) for statements such as, “I have so much in life to be thankful for” and “As I get older I find myself more able to appreciate the people, events, and situations that have been part of my life history.” Higher scores indicated a higher propensity to perceive gratitude in daily experiences. The Cronbach *α* for this measurement was high (Cronbach *α*=.84), indicating high internal consistency [28].

#### Quality of Life

Quality of life was measured using the 4-item self-reported Centers for Disease Control Healthy Days Survey [29,30]. An additional question assessing overall quality of life was also added: “How would you rate your overall quality of life?” Patients were asked to rate their overall quality of life and quality of general health using a 5-point Likert scale, ranging from 1 (*poor*) to 5 (*excellent*), and indicate the number of days out of the previous 30 days that they experienced either one or both: poor mental or physical health. A higher number of unhealthy days indicated a lower quality of life.

#### Self-efficacy of Sustained Sobriety

Self-reported measurements of patients’ confidence in their ability to stay sober comprised an adapted form of the *Brief Situational Confidence Questionnaire* to create a sobriety self-efficacy scale [31]. Sample questions included, “I would be able to resist using alcohol or drugs right now if I were physically uncomfortable [eg, feeling sick, headache, and in pain]*”* and “I would be able to resist using alcohol or drugs right now if someone I cared about offered it to me [eg, a good friend at a gathering or a spouse at home]” [31].

The 7-point Likert response categories were reworded from 1 (*not confident at all*) to 7 (*totally confident*) to 1 (*strongly disagree*) to 7 *(strongly agree*) to maintain consistency across the different scales. Initial interitem correlations and *α* values indicated that in comparison with the other questions, the original Brief Situational Confidence Questionnaire question 5, “I could probably go back to social drinking or other moderate drug use if I wanted to,” did not adequately add to the measure of sobriety self-efficacy. After discussing the inventory with the DCSs, it was determined that multiple patients were unable or unwilling to answer item 5 when prompted. This question was likely uniquely difficult for this population, given the abstinence-based focus of HBFF’s programming and the consistent message during treatment that no amount of substance use is safe. This item was removed shortly after data collection began in 2020. After the removal of item 5, the adapted scale of sobriety self-efficacy showed high internal consistency (Cronbach *α*=.89).

#### Peer Group Support Engagement

Self-reports of engaging in peer group support were measured by 1 item adapted from the Alcoholic Anonymous Involvement Scale, which asked respondents, “About how often have you been attending 12-step/peer support/mutual aid group meetings since you were discharged?*”* [32]. Adaptations were made to include peer support groups other than alcoholics anonymous. Participants answered using a 6-point ordinal scale: *daily, ≥4 times per week, 1-3 times per week, 2-4 times per month, once a month or less,* or *never.*

#### Parenting Stressors

The 20-item Parenting Daily Hassles Scale was used to measure the frequency and intensity (or impact) of common daily parenting or caregiver stressors [33,34]. Respondents were also asked to rate the frequency of occurrence of experienced hassles on a 4-point Likert scale, ranging from 1 (*rarely*) to 4 (*constantly*). In addition, *parents* was changed to *caretakers* for inclusion of nontraditional caregivers. Sample questions included, “For the past 6 months, how often have you found yourself continually cleaning up messes of toys or food” and “The kids are constantly underfoot, interfering with other chores.” The internal consistency was excellent (Cronbach *α*=.91).

Finally, 2 questions were added to capture participants who had to manage homeschooling because of the pandemic: (1) “Since January 2020, have you had to manage homeschooling for children under 18 due to the pandemic?” which participants answered *yes* or *no* and “During any of the following months, did you have to manage homeschooling for children?” where participants selected all months in which they homeschooled.

#### History of Parental (Family of Origin) Alcohol or Drug Use

We used the 6-item modified Children of Alcoholics Screening Test to assess exposure to parental alcoholism [35]. Participants were able to opt out of this question if it did not apply. Questions were modified to include exposure to parental drug use, such as *drinking or drug problem* or *drunk or high*. In addition, the gendered language in item 3 was changed from *he or she* to *they* for gender inclusion. Respondents were asked *yes* or *no* questions related to past experiences with their parents and alcohol or drugs. Questions included, “Have you ever thought that one of your parents had a drinking or drug problem?” and “Have you ever heard your parents fight when one of them was drunk or high?” The internal consistency of this scale was a Cronbach *α* of .90.

#### Lifetime Exposure to Family and Intimate Partner Violence

The 3-item survey adapted from the study by Easton et al [36] based on the Revised Conflict Tactics Scale was used to measure the lifetime prevalence of family and intimate partner violence [37]. Participants answered *yes* or *no* to questions related to their lifetime exposure to childhood physical and sexual violence in addition to a history of perpetrating or experiencing intimate partner violence. For the purposes of this study, we modified 2 questions to parse out those with exposure to physical and sexual violence. For example, item 3 (“As a child, were you ever physically or sexually hurt by a parent, family member, friend of the family, or some other adult? [eg, slapped, pushed, punched, beat up, or sexually abused]” was changed to, “As a child, were you every sexually hurt by a parent, family member, friend of the family, or some other adult? [eg, sexually abused]” and “As a child, were you ever physically hurt by a parent, family member, friend of the family, or some other adult? [eg, slapped, pushed, punched, or beat up]*.*” Other questions included, “In your lifetime, have you been in a fight with a spouse or partner in which you were physically hurt? [eg, slapped, pushed, punched, beat up, or sexually assaulted].” The internal consistency of the scale was a Cronbach *α* of .53. In addition, we added 1 question to measure the respondent-perceived impact of violence exposure on a 10-point Likert scale (ie, 1=*not at all* to 10=*enormous daily impact*).

#### Drug and Alcohol Use

To measure self-reported substance use duration and severity during the study period, we used the modified Form-90 Quick Drinking Assessment (Form-90-AQ) [38]. The Form-90-AQ was developed as a brief assessment tool to determine an individual’s alcohol use during a discrete period leading up to the present day [38]. Questions were modified to improve clarity when given over the phone (eg, including each participant’s specific period as well as the number of days in the period in every question, rather than only in the initial prompt). Sample questions included, “Have you used any alcohol since discharge or your last survey on [last survey or discharge date], a period of [number of days between today and last survey or discharge]?” and “On those days when you did drink, how much did you have to drink on average?” Next, the question included in the Form-90-AQ about binge drinking was updated to reflect the National Institute on Alcohol Abuse and Alcoholism’s (NIAAA’s) most recent recommendation for the definition of the concept, from 6 to 5 drinks (eg, “Of those days on which you drank, on how many days did you have five or more drinks?”)*.* In addition, a question about blacking out from alcohol use was added (eg, “On those days on which you drank, on how many days did you drink so much that you *blacked out* or couldn’t remember?”) [39]. Finally, some questions were adapted to ask about other substance use (eg, *“*Have you used any drugs or alcohol since your last survey on [last survey date]” and “Have you used any drugs, not including tobacco/nicotine, since your last survey on [last survey date], a period of [number of days between today and last survey]?”

### Statistical Analyses

Analyses were conducted using SPSS statistics (*version* 28; IBM Corp) [40]. Chi-square tests of independence and 1-way ANOVA were performed to examine the relationships between the group format and baseline participant characteristics.

## Results

### Sample Characteristics

**Figure 2:**
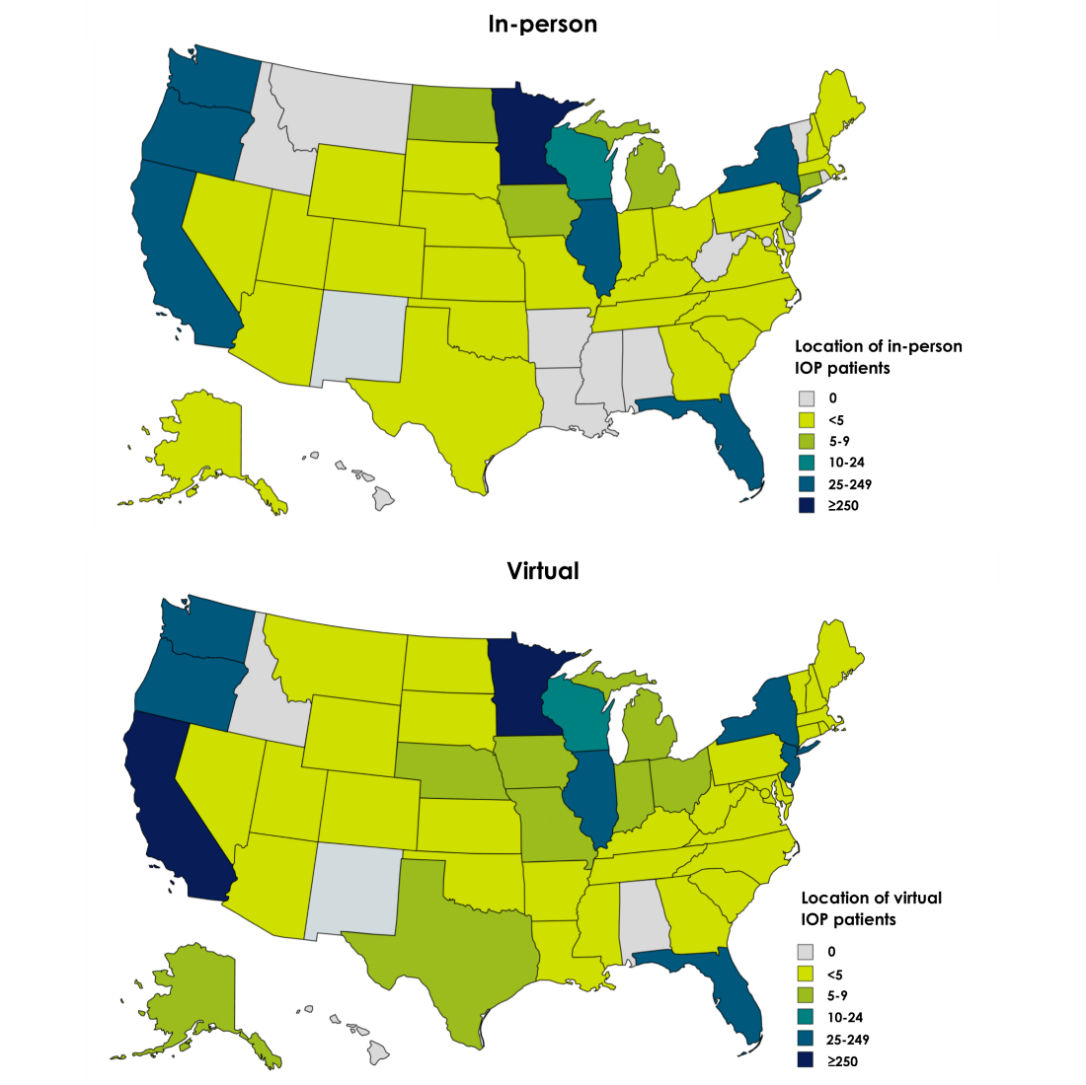
Geographic distribution of participants by home state. IOP: intensive outpatient program.

The sample characteristics are reported by IOP setting in Multimedia Appendix 1. Most patients were White (3296/3642, 90.49%) and men (2258/3642, 62%), with a mean age of 39.1 (SD 13.5) years. Approximately 37.97% (1383/3642) of the patients were diagnosed with 2 or more active substance use diagnoses, and the vast majority (3519/3642, 96.62%) accessed treatment through insurance. The national spread of patients by home state is shown displayed in Figure 2. Patients from a greater number of states attended VIOP (37 states: in-person and 46 states+Washington DC: internet-based), suggesting increased accessibility to care. Data were also considered according to the developmental stages presented in Multimedia Appendix 2, ranging from emerging adulthood to late adulthood to better understand how an individual’s age may impact participation and engagement in different modalities of IOP.

### Bivariate Group Comparisons: IOP Setting

At the beginning of the transition to web-based services in early 2020, a small proportion of patients were discharged before treatment completion when they elected not to switch to VIOP or when they stopped attending the sessions shortly after the change in treatment modality occurred. Patients who were discharged within 14 days of a group switching to internet-based were significantly more likely than those in the hybrid or web-based-only groups to be men (*χ*^2^_2_=8.7; *P*<.05), to be younger (*χ*^2^_4_=24.7; *P*<.001), and to have obtained a lower level of education (*χ*^2^_4_=12.7; *P*<.05). There were no significant differences between these groups in the proportions of White, Hispanic, or Latinx individuals; employed full time or part time; or diagnosed with one or more SUDs.

Key features included differences in biological sex distribution, where participants in the hybrid group were significantly more likely to be men, whereas those in the internet-based group were more likely to be women. Significant differences were observed in age distributions. A greater number of individuals aged 18-25 years participated in the hybrid, and a greater number of individuals aged 45-64 years participated in the internet-based-only IOP. Regarding marital status, those in the hybrid group were significantly more likely to be single, whereas those in the web-based-only programming were more likely to be divorced, separated, or widowed. Individuals in hybrid programming had significantly longer lengths of stay and were significantly less likely to be discharged against staff or medical advice than individuals in both in-person and internet-only IOP. No significant differences were detected between formats by race, ethnicity, employment status, education level, or whether the participants presented with multiple SUDs. Similarly, no differences emerged in the type of SUD except for cocaine use disorder, where a significantly higher proportion of participants in the in-person group and a lower proportion in the internet-only group were diagnosed with a cocaine use disorder.

### Developmental Stages

More differences emerged when examining the relationship between developmental stage and baseline participant characteristics, primarily driven by the emerging adults (those aged 18-25 years at treatment entry) in the sample (full results in Multimedia Appendix 2). Emerging adults were significantly more likely to participate in hybrid programming and less likely to participate in the web-based-only IOP. In terms of biological sex distribution, emerging and early adults were significantly more likely to be men, whereas middle-aged adults were more likely to be women. Owing to the preponderance of White participants in the sample, the race variable was collapsed to compare White with non-White participants (with full self-reported identification reported in Multimedia Appendix 1). Emerging adults were significantly more likely to be non-White, whereas middle-aged adults were significantly more likely to be White. Differences also emerged related to programming, with those aged 18-25 years engaging in treatment for a significantly longer period than older participants while also being more likely to be discharged against staff or medical advice. Emerging adults were less likely to be diagnosed with an alcohol use disorder and more likely to be diagnosed with all other SUDs, except for inhalant and other psychoactive disorders. Finally, emerging adults were significantly more likely to be diagnosed with multiple SUDs.

Regarding baseline clinical and functional measurements (Table 2), no differences emerged between the formats. Missing values reflect a delay in our ability to collect baseline and 1-month data from the in-person and hybrid groups.

**Table 2:** Baseline measurements of participants in intensive outpatient program.

Average baseline measurement scores	In-person only (n=957), mean (SD)	Hybrid (n=541), mean (SD)	Internet only (n=2144), mean (SD)	Overall (N=3642); missing, n (%)	Overall, *F* test (*df*)	*P* value
Psychological well-being	N/A^a^	N/A	42.81 (9.24)	2845 (78.12)	N/A	N/A
Financial Well-being Scale	N/A	N/A	49.37 (6.06)	2824 (77.54)	N/A	N/A
Gratitude Questionnaire—6-item form	N/A	N/A	34.36 (6.39)	2824 (77.54)	N/A	N/A
Quality of life	N/A	N/A	3.69 (0.88)	2810 (77.16)	N/A	N/A
Sobriety self-efficacy	N/A	N/A	5.57 (1.36)	2831 (77.73)	N/A	N/A
History of family violence	N/A	N/A	0.79 (0.94)	2859 (78.50)	N/A	N/A
History of parental substance use	N/A	N/A	2.06 (2.27)	2860 (78.53)	N/A	N/A
Frequency of parenting stressors	N/A	N/A	37.29 (10.33)	3464 (95.11)	N/A	N/A
Patient Health Questionnaire-9	6.18 (5.27)	5.73 (4.92)	6.48 (5.47)	2710 (74.41)	0.96 (2, 929)	.38
General Anxiety Disorder-7	6.20 (5.18)	5.95 (5.95)	6.85 (5.18)	2560 (70.30)	2.51 (2, 1079)	.08
Commitment to Sobriety Scale	27.01 (2.95)	27.04 (3.36)	27.14 (3.40)	2493 (68.45)	0.20 (2, 1146)	.82
DSQ^b^—desire and intention to use	1.72 (1.08)	1.70 (1.08)	1.74 (1.03)	1685 (46.27)	0.15 (2, 1954)	.86
DSQ—negative reinforcement	1.92 (1.37)	1.85 (1.11)	1.94 (1.23)	1685 (46.27)	0.52 (2, 1954)	.59

^a^N/A: not applicable.

^b^DSQ: Desire for Speed Questionnaire.

## Discussion

### Principal Findings

The VIOP study represents an important advancement in expanding our understanding of the role of telehealth in alcohol and substance use addiction treatment, providing richer insight into whether comparable care can be delivered through the internet.

At treatment entry, adults were similar across most demographic and substance use variables for all formats of IOP. Similarly, average scores on clinical inventories (eg, PHQ-9 and GAD-7) at baseline did not differ significantly by delivery setting, illustrating similar levels of psychiatric symptoms and physical cravings, regardless of the IOP delivery setting and timing of treatment in relation to the pandemic. This supports the literature on the use of web-based methods for the treatment of mental health symptoms and also shows that this treatment modality is viable for substance use populations as well. One notable difference emerged: individuals who participated in hybrid programming stayed in treatment significantly longer and were discharged against staff advice at a lower rate than those in traditional (in-person) or internet-based-only programming. Future publications will assess whether hybrid settings improved patient outcomes over and beyond those who received care in 1 setting only or whether it was a reflection of the extra support needed during a time of significant disruption in individuals’ lives associated with the onset of the COVID-19 pandemic.

More differences emerged when comparing participants across developmental stages. Emerging adults (aged 18-25 years) in IOP showed consistent differences across a variety of demographic variables than those in early adulthood, mid-adulthood, and late adulthood and were more likely than patients at other developmental stages to present with multiple co-occurring SUDs, have longer episodes of care, and discharge early despite recommendations by program staff to continue treatment. These results suggest that patients in emerging adulthood have unique needs over and beyond those in older adult stages.

### Strengths and Limitations

There are strengths and limitations to the use of ROM data. As these explorations are not bound by a clinical trial, there are inherent measurement errors that may affect the data, such as instances of inaccurate manual data entry, missed measurement scores, or diversity of standard measurements used in initial diagnostic assessments across clinical service departments and disciplines (ie, solely alcohol and substance use treatment vs co-occurring treatment). As a result, mental health diagnoses outside of substance and alcohol use disorders were not consistently documented and had to be excluded from the analyses. As data collection began in May 2020, much of our baseline data were pulled retrospectively through electronic health records, and in-person and hybrid patients missed some baseline measurements and what would have been their 1-month follow-up. Although not ideal, the remaining baseline measurements and 3-month follow-up can be compared, and any notable differences will inform future research. Furthermore, findings will be limited by the predominance of White, non-Hispanic men in our sample and therefore should not be generalized to patient populations’ representative of minority and marginalized persons. Owing to the lack of randomization, our results do not allow for causal associations. However, these findings will provide an ecologically valid examination of web-based care in an existing health care system that is relevant and informative to other health care systems providing alcohol and substance use addiction treatment. Indeed, clinically efficacious explorations are inherently advantageous, and ecologic examinations may offer richer insight and practical implications in the real-world day-to-day lived experience of patients undergoing alcohol and substance use treatment.

### Conclusions

Future findings hope to inform the effective development of data-driven benchmarks and protocols for routine outcome data practice. Investigations may also leverage these data to identify the patients for whom and circumstances under which telehealth is most efficacious, as these services are integrated into the standard of care for addiction treatment and recovery.

## Appendix

Multimedia Appendix 1 Baseline demographic characteristics of patients enrolled in intensive outpatient program (IOP) in 2020.

Multimedia Appendix 2 Baseline characteristics of intensive outpatient program (IOP) patients by developmental stage in 2020 (N=3642).

## Abbreviations

CSS: Commitment to Sobriety Scale
DCS: data collection specialist
GAD-7: General Anxiety Disorder-7
HBFF: Hazelden Betty Ford Foundation
IOP: intensive outpatient program
PHQ-9: Patient Health Questionnaire-9
ROM: routine outcome monitoring
SUD: substance use disorder
VIOP: virtual intensive outpatient program
