# Supplementary material for: In-Person Versus Telehealth Setting for the Delivery of Substance Use Disorder Treatment: Ecologically Valid Comparison Study
Source: JMIR Form Res. 2022 Apr 4;6(4):e34408. doi: 10.2196/34408 (PMC9016509; doi:10.2196/34408)
Supplement: Multimedia Appendix 1 [file formative_v6i4e34408_app1.docx]

**Multimedia Appendix 1.** Baseline demographic characteristics of patients enrolled in intensive outpatient program (IOP) in 2020.

| Characteristics | | In-person only (n=957) | Hybrid (n=541) | Virtual only (n=2144) | | Overall (N=3642) | *F* (df) | Overall chi-square (df) | *P* value |
| --- | --- | --- | --- | --- | --- | --- | --- | --- | --- |
| **Biological sex (n=3642), n (%)** | | | | | | | —^a^ | 8.46 (2) | <.05 |
|  | Male | 595 (62.2) | 364 (67.4) | 1299 (60.6) | | 2258 (62) |  |  |  |
|  | Nonbinary | — | — | — | | 2 (0.1) |  |  |  |
|  | Missing | — | — | — | | 0 (0) |  |  |  |
| **Age (years; n=3642), n (%)** | | | | | | | — | 26.83 (6) | <.001 |
|  | 18-25 | 183 (19.1) | 139 (25.7) | 382 (17.9) | | 704 (19.4) |  |  |  |
|  | 26-44 | 434 (45.4) | 245 (45.3) | 935 (43.8) | | 1614 (44.4) |  |  |  |
|  | 45-64 | 310 (32.4) | 149 (27.5) | 769 (36.) | | 1228 (33.8) |  |  |  |
|  | ≥65 | 30 (3.1) | 8 (1.5) | 51 (2.4) | | 89 (2.4) |  |  |  |
|  | Missing | — | — | — | | 0 (0) |  |  |  |
| **Race (n=3609), n (%)** | | | | | | | — | 0.08 (2) | .96 |
|  | American Indian or Alaskan Native | 4 (0.4) | 3 (0.6) | 14 (0.7) | | 21 (0.6) |  |  |  |
|  | Asian or Asian American | 15 (1.6) | 14 (2.6) | 25 (1.2) | | 54 (1.5) |  |  |  |
|  | Black or African American | 21 (2.2) | 17 (3.1) | 42 (2) | | 80 (2.2) |  |  |  |
|  | Native Hawaiian or other Pacific Islander | 2 (0.2) | 1 (0.2) | 3 (0.1) | | 6 (0.2) |  |  |  |
|  | White | 867 (91.3) | 491 (90.9) | 1938 (91.5) | | 3296 (91.3) |  |  |  |
|  | Biracial or multiracial (>2 races) | 12 (1.3) | 5 (0.9) | 36 (1.7) | | 53 (1.5) |  |  |  |
|  | Other | 29 (3.1) | 9 (1.7) | 61 (2.9) | | 99 (2.7) |  |  |  |
|  | Missing | — | — | — | | 33 (0.01) |  |  |  |
| **Ethnicity (n=3469), n (%)** | | | | | | | — | 0.84 (2) | .66 |
|  | Not Hispanic or Latinx or Spanish origin | 873 (95) | 502 (95.1) | 1907 (94.3) | 3282 (94.6) | |  |  |  |
|  | Hispanic or Latinx or Spanish origin | 46 (5) | 26 (4.9) | 115 (5.7) | 187 (5.4) | |  |  |  |
|  | Missing | — | — | — | 173 (4.8) | |  |  |  |
| **Marital status (n=3602), n (%)** | | | | | | | — | 20.47 (4) | <.001 |
|  | Single, never married | 423 (44.6) | 271 (50.3) | 871 (41.2) | 1565 (43.4) | |  |  |  |
|  | Cohabiting | 18 (1.9) | 9 (1.7) | 52 (2.5) | 79 (2.2) | |  |  |  |
|  | Married or life partner | 382 (40.3) | 213 (39.5) | 895 (42.3) | 1490 (41.4) | |  |  |  |
|  | Married but separated | 34 (3.6) | 12 (2.2) | 70 (3.3) | 116 (3.2) | |  |  |  |
|  | Divorced | 78 (8.2) | 31 (5.8) | 210 (9.9) | 319 (8.9) | |  |  |  |
|  | Widowed | 13 (1.4) | 3 (0.6) | 17 (0.8) | 33 (0.9) | |  |  |  |
|  | Missing | — | — | — | 40 (1.1) | |  |  |  |
| **Employment status** **(n=3556), n (%)** | | | | | | | — | 2.51 (2) | .29 |
|  | Full-time employment/self-employed | 555 (58) | 295 (54.5) | 1268 (59.1) | 2118 (58.2) | |  |  |  |
|  | Part-time employment | 38 (4) | 24 (4.4) | 83 (3.9) | 145 (4) | |  |  |  |
|  | Home and family manager, student (full time or part time), or retired | 100 (10.5) | 60 (11) | 188 (8.9) | 348 (13.1) | |  |  |  |
|  | Unemployment, actively seeking a job | 53 (5.5) | 24 (4.4) | 102 (4.8) | 179 (4.9) | |  |  |  |
|  | Unemployment, not seeking a job | 194 (20.3) | 127 (23.5) | 433 (20.2) | 754 (21.2) | |  |  |  |
|  | Missing | — | — | — | 86 (2.4) | |  |  |  |
| **Education level** **(n=2529), n (%)** | | | | | | | — | 7.86 (4)^b^ | .10 |
|  | Some high school or less, no diploma | 14 (2) | 7 (1.8) | 40 (2.8) | 61 (2.4) | |  |  |  |
|  | High school diploma or equivalent (general educational development) | 87 (12.5) | 64 (16.4) | 225 (15.6) | 376 (14.9) | |  |  |  |
|  | Some college, no degree | 132 (19) | 100 (25.6) | 324 (22.5) | 556 (22) | |  |  |  |
|  | Associate degree/Vo-Tech | 45 (6.5) | 31 (7.9) | 121 (8.4) | 197 (7.8) | |  |  |  |
|  | College graduate/bachelor’s degree | 302 (43.5) | 141 (36.1) | 527 (36.5) | 970 (38.4) | |  |  |  |
|  | Graduate/professional degree | 115 (16.5) | 48 (12.3) | 206 (14.3) | 389 (14.6) | |  |  |  |
|  | Missing | — | — | — | 1113 (30.6) | |  |  |  |
| **Length of stay in IOP (n=3642), mean (SD)** | | | | | | | 48.67 (2, 3639) | — | <.001 |
|  | Average length of stay (days) | 47.99 (33.43) | 77.19 (48.85) | 47.62 (34.41) | 52.11 (38.13) | |  |  |  |
|  | Missing | — | — | — | 0 (0) | |  |  |  |
| **Discharged against staff advice (n=3642), n (%)** | | | | | | | — | 10.60 (2) | <.01 |
|  | Yes | 181 (18.9) | 71 (13.1) | 407 (19) | 659 (18.1) | |  |  |  |
|  | No | 776 (81.1) | 470 (86.9) | 1737 (81) | 2983 (81.9) | |  |  |  |
|  | Missing | — | — | — | 0 (0) | |  |  |  |
| **Used insurance for services (n=3642), n (%)** | | | | | | | — | 7.10 (2) | <.05 |
|  | Yes | 912 (95.3) | 524 (96.9) | 2083 (97.2) | | 3519 (96.6) |  |  |  |
|  | Self-pay | 45 (4.7) | 17 (3.1) | 61 (2.8) | | 123 (3.4) |  |  |  |
|  | Missing | — | — | — | | 0 (0) |  |  |  |
| **Active SUD^c^ diagnosis (n=3642), n (%)** | | | | | | | | | |
|  | Alcohol use disorder | 792 (82.8) | 448 (82.8) | 1815 (84.7) | | 3055 (83.9) | — | 2.30 (2) | .32 |
|  | Cannabis use disorder | 225 (23.5) | 151 (27.9) | 502 (23.4) | | 878 (24.1) | — | 5.03 (2) | .08 |
|  | Cocaine use disorder | 109 (11.4) | 59 (10.9) | 163 (7.6) | | 331 (9.1) | — | 14.03 (2) | <.01 |
|  | Hallucinogen use disorder | 9 (0.9) | 9 (1.7) | 15 (0.7) | | 33 (0.9) | — | 4.49 (2) | .11 |
|  | Inhalant use disorder | 3 (0.3) | 2 (0.4) | 6 (0.3) | | 11 (0.3) | — | 0.12 (2) | .94 |
|  | Opioid use disorder | 128 (13.4) | 89 (16.5) | 273 (12.7) | | 490 (13.5) | — | 5.14 (2) | .08 |
|  | Sedative use disorder | 104 (10.9) | 55 (10.2) | 246 (11.5) | | 405 (11.1) | — | 0.83 (2) | .66 |
|  | Other stimulant use disorder | 124 (13) | 63 (11.6) | 254 (11.8) | | 441 (12.1) | — | 0.90 (2) | .64 |
|  | Other psychoactive substance use disorder | 11 (1.1) | 7 (1.3) | 34 (1.6) | | 52 (1.4) | — | 0.98 (2) | .61 |
|  | Missing | — | — | — | | 0 (0) | — | — | — |
| **Number of co-occurring SUD diagnoses (n=3642), n (%)** | | | | | | | — | 11.37 (8) | .18 |
|  | 1 | 577 (60.3) | 327 (60.4) | 1355 (63.2) | | 2259 (62) |  |  |  |
|  | 2 | 251 (26.2) | 121 (22.4) | 516 (24.1) | | 888 (24.4) |  |  |  |
|  | 3 | 99 (10.3) | 67 (12.4) | 197 (9.2) | | 363 (10) |  |  |  |
|  | 4 | 23 (2.4) | 18 (3.3) | 53 (2.5) | | 94 (2.6) |  |  |  |
|  | 5 or more | 7 (0.7) | 8 (1.5) | 23 (1.1) | | 38 (1) |  |  |  |
|  | Missing | — | — | — | | 0 (0) |  |  |  |

^a^ Not applicable

^b^Variables where categories were collapsed into 2 or 3 levels to test for group differences because of small cell sizes.

^c^SUD: substance use disorder.

This is a Multimedia Appendix to a full manuscript published in JMIR Formative Research. For full copyright and citation information see http://dx.doi.org/10.2196/jmir.34408
