# Supplementary material for: In-Person Versus Telehealth Setting for the Delivery of Substance Use Disorder Treatment: Ecologically Valid Comparison Study
Source: JMIR Form Res. 2022 Apr 4;6(4):e34408. doi: 10.2196/34408 (PMC9016509; doi:10.2196/34408)
Supplement: Multimedia Appendix 2 [file formative_v6i4e34408_app2.docx]

**Multimedia Appendix 2.** Baseline characteristics of intensive outpatient program (IOP) patients by developmental stage in 2020 (N=3642).

| Characteristics | | Emerging adulthood (aged 18-25 years; n=704) | Early adulthood (aged 26-44 years; n=1614) | Middle adulthood (aged 45-64 years, n=2144) | Late adulthood (aged ≥65 years; n=96) | *F* (df) | Overall chi-square (df) | *P* value |
| --- | --- | --- | --- | --- | --- | --- | --- | --- |
| **Type of treatment setting (n=3642), n (%)** | | | | | | —^a^ | 26.83 (6) | <.001 |
|  | In-person | 183 (25.7) | 434 (26.9) | 310 (25.2) | 30 (33.7) |  |  |  |
|  | Hybrid | 139 (19.5) | 245 (15.2) | 149 (12.1) | 8 (9) |  |  |  |
|  | Virtual | 389 (54.7) | 935 (57.9) | 769 (62.6) | 51 (57.3) |  |  |  |
|  | Missing | — | — | — | 0 (0) |  |  |  |
| **Biological sex** **(n=3642), n (%)** | | | | | | — | 40.46 (3) | <.001 |
|  | Male | 485 (68.2) | 1043 (64.7) | 679 (55.3) | 51 (57.3) |  |  |  |
|  | Nonbinary | — | — | — | 2 (0.1) |  |  |  |
| **Race (n=3609), n (%)** | | | | | | — | 15.81 (3)^b^ | <.001 |
|  | American Indian or Alaskan Native | 6 (0.8) | 8 (0.5) | 7 (0.6) | 0 (0) |  |  |  |
|  | Asian or Asian American | 7 (1) | 21 (1.3) | 8 (0.7) | 0 (0) |  |  |  |
|  | Black or African American | 19 (2.7) | 39 (2.4) | 20 (1.6) | 2 (2.2) |  |  |  |
|  | Native Hawaiian or other Pacific Islander | 1 (0.1) | 4 (0.2) | 1 (0.1) | 0 (0) |  |  |  |
|  | White | 623 (87.6) | 1451 (89.9) | 1138 (92.7) | 84 (94.4) |  |  |  |
|  | Biracial or multiracial (>2 races) | 23 (3.2) | 21 (1.3) | 9 (0.7) | 0 (0) |  |  |  |
|  | Other | 26 (3.6) | 54 (3.4) | 34 (2.8) | 3 (3.3) |  |  |  |
|  | Missing | — | — | — | 26 (0.7) |  |  |  |
| **Ethnicity (n=3469), n (%)** | | | | | | — | 4.40 (3) | .22 |
|  | Hispanic or Latino or Spanish origin | 42 (6.2) | 89 (5.8) | 54 (4.6) | 2 (2.3) |  |  |  |
|  | Not Hispanic or Latino or Spanish origin | 633 (93.8) | 1447 (94.2) | 1118 (95.4) | 84 (97.7) |  |  |  |
|  | Missing | — | — | — | 173 (4.8) |  |  |  |
| **Length of stay in IOP (n=3642), mean (SD)** | | | | | | 13.51 (3, 3638) | — | <.001 |
|  | Average length of stay (days) | 59.96 (46.33) | 51.23 (35.86) | 49.02 (34.89) | 51.77 (37.32) |  |  |  |
|  | Missing | — | — | — | 0 (0) |  |  |  |
| **Discharged against staff advice (n=3642), n (%)** | | | | | | — | 13.32 (3) | <.01 |
|  | Yes | 152 (21.4) | 306 (19) | 189 (15.4) | 12 (13.5) |  |  |  |
|  | No | 559 (78.6) | 1308 (81) | 1039 (85.6) | 77 (86.5) |  |  |  |
|  | Missing | — | — | — | 0 (0) |  |  |  |
| **Active SUD^c^ diagnosis (n=3642), n (%)** | | | | | | | | |
|  | Alcohol use disorder | 485 (68.2) | 1357 (84.1) | 1133 (92.3) | 80 (89.9) | — | 195.34 (3) | <.001 |
|  | Cannabis use disorder | 427 (60.1) | 347 (21.5) | 99 (8.1) | 5 (5.6) | — | 697.65 (3) | <.001 |
|  | Cocaine use disorder | 117 (16.5) | 161 (10) | 53 (4.3) | 0 (0) | — | 90.99 (3) | <.001 |
|  | Hallucinogen use disorder | 30 (4.2) | 1 (0.1) | 2 (0.2) | 0 (0) | — | 108.11 (3) | <.001 |
|  | Inhalant use disorder | 4 (0.6) | 4 (0.3) | 2 (0.2) | 0 (0) | — | 2.67 (3) | .45 |
|  | Opioid use disorder | 195 (27.4) | 203 (12.6) | 85 (6.9) | 6 (6.7) | — | 168.57 (3) | <.001 |
|  | Sedative use disorder | 144 (20.3) | 167 (10.3) | 84 (6.8) | 10 (11.2) | — | 83.74 (3) | <.001 |
|  | Other stimulant use disorder | 144 (20.3) | 210 (13) | 86 (7) | 1 (1.1) | — | 85.72 (3) | <.001 |
|  | Other psychoactive substance use disorder | 12 (1.7) | 27 (1.7) | 13 (1.1) | 0 (0) | — | 3.51 (3) | .32 |
|  | Missing | — | — | — | 0 (0) | — | — | — |
| **Prevalence of co-occurring SUD diagnoses (n=3642), n (%)** | | | | | | — | 453.55 (3) | <.001 |
|  | Single | 224 (31.5) | 989 (61.3) | 968 (78.8) | 78 (87.6) |  |  |  |
|  | 2 or more | 487 (68.5) | 625 (38.7) | 260 (21.2) | 11 (12.4) |  |  |  |
|  | Missing | — | — | — | 0 (0) |  |  |  |

^a^ Not applicable

^b^Variables where categories were collapsed into 2 or 3 levels to test for group differences because of small cell sizes.

^c^SUD: substance use disorder.

This is a Multimedia Appendix to a full manuscript published in JMIR Formative Research. For full copyright and citation information see http://dx.doi.org/10.2196/jmir.34408
